# Comparative Evaluation of the Vector Competence of Four South American Populations of the *Rhipicephalus sanguineus* Group for the Bacterium *Ehrlichia canis*, the Agent of Canine Monocytic Ehrlichiosis

**DOI:** 10.1371/journal.pone.0139386

**Published:** 2015-09-28

**Authors:** Jonas Moraes-Filho, Felipe S. Krawczak, Francisco B. Costa, João Fábio Soares, Marcelo B. Labruna

**Affiliations:** 1 Department of Preventive Veterinary Medicine and Animal Health, Faculty of Veterinary Medicine, University of São Paulo, São Paulo, SP, Brazil; 2 Universidade de Cruz Alta - UNICRUZ, Cruz Alta, RS, Brazil; University of Minnesota, UNITED STATES

## Abstract

This study compared the vector competence of four populations of *Rhipicephalus sanguineus* group ticks for the bacterium *Ehrlichia canis*, the agent of canine monocytic ehrlichiosis (CME). Ticks (larvae and nymphs) from the four populations—one from São Paulo state, southeastern Brazil (BSP), one from Rio Grande do Sul state, southern Brazil (BRS), one from Argentina (ARG), and one from Uruguay (URU)–were exposed to *E*. *canis* infection by feeding on dogs that were experimentally infected with *E*. *canis*. Engorged ticks (larvae and nymphs) were allowed to molt to nymphs and adults, respectively, which were tested by molecular analysis (*E*. *canis*-specific PCR assay) and used to infest naïve dogs. Through infestation of adult ticks on naïve dogs, after nymphal acquisition feeding on *E*. *canis*-infected dogs, only the BSP population was shown to be competent vectors of *E*. *canis*, i.e., only the dogs infested with BSP adult ticks developed clinical illness, seroconverted to *E*. *canis*, and yielded *E*. *canis* DNA by PCR. This result, demonstrated by two independent replications, is congruent with epidemiological data, since BSP ticks were derived from São Paulo state, Brazil, where CME is highly endemic. On the other hand, BRS, ARG, and URU ticks were derived from a geographical region (South America southern cone) where CME has never been properly documented. Molecular analysis of unfed adults at 30 days post molting support these transmission results, since none of the BRS, ARG, and URU ticks were PCR positive, whereas 1% of the BSP nymphs and 31.8% of the BSP adults contained *E*. *canis* DNA. We conclude that the absence or scarcity of cases of CME due to *E*. *canis* in the South America southern cone is a result of vector incompetence of the *R*. *sanguineus* group ticks that prevail on dogs in this part of South America.

## Introduction

The *Rhipicephalus sanguineus* complex is a group of at least 12 morphologically closely related species, including *Rhipicephalus sanguineus* sensu stricto (s.s.) [1]. Until the end of the 20^th^ century, the taxon *R*. *sanguineus* was thought to represent a single tick species with a nearly cosmopolitan distribution, mainly associated with domestic dogs [2,3]. During the last 10 years, a number of studies based on molecular [4,5,6,7], biological [4], and morphological [8] analyses revealed that at least two distinct species have been considered under the taxon *R*. *sanguineus* in Latin America. Moraes-Filho et al. [6] assigned these two species as ‘temperate’ and ‘tropical’, the former restricted to the southern cone of South America (Uruguay, Argentina, Chile, and the southernmost state of Brazil), and the later encompassing the rest of Latin America, from Mexico to Brazil. This distribution, based on genetic analysis, was corroborated by subsequent data [7]. Similarly to the current situation of Latin America, recent genetic studies showed that the taxon *R*. *sanguineus* s.s. was also applied to distinct genospecies on other continents [6,9,10,11], also corroborated by biological analysis [9]. In view of this unequivocally taxonomic problem, Nava et al. [1] recommended that, currently, it is not possible to assign the specific name *R*. *sanguineus* s.s. to any tick population of the world. Until this issue is not solved, the term “*R*. *sanguineus* group” should be employed instead of *R*. *sanguineus* s.s., in future studies [1]. We have adopted this recommendation in the present study.

The bacterium *Ehrlichia canis* is the etiological agent of canine monocytic ehrlichiosis (CME), a tick-borne disease of domestic dogs in many parts of the world [12], including South America [13]. Ticks of the *R*. *sanguineus* group are primary vectors of *E*. *canis* to dogs [14,15,16,17]. Because there is no transovarial transmission of *E*. *canis* in *R*. *sanguineus* group [14], dogs acquire the infection when infested by an infected nymph or adult tick that had acquired the infection in a previous developmental stage (transstadial transmission) or the same stage (intrastadial transmission) by feeding on an acutely or chronically infected dog [17,18].

According to a recent review [19] and subsequent studies [20,21,22,23], molecular detection of *E*. *canis* DNA has been achieved in blood samples from naturally infected dogs of all regions of Brazil where it has been attempted, except for the southernmost state, Rio Grande do Sul [24]. In addition, while canine seroprevalence values within 30–75% for *E*. *canis* have been reported in random samples of dogs from different regions of Brazil [22,25,26,27,28,29], studies in Rio Grande do Sul have reported seroprevalence values always below 5% [24,30,31,32], despite local abundance of *R*. *sanguineus* group ticks on dogs [33,34]. Because Rio Grande do Sul is the only part of Brazil where the ‘temperate species’ of the *R*. *sanguineus* group has been detected, in contrast to the widespread distribution of the ‘tropical species’ in the remaining regions of the country [6], we hypothesize that the low prevalence of *E*. *canis* in Rio Grande do Sul is related to possible vector incompetence of *R*. *sanguineus* group ticks from this region. Therefore, the present study evaluated a comparative analysis of the vector competence of different populations of *R*. *sanguineus* group, including one from São Paulo, southeastern Brazil, where CME is highly endemic [19], and one from Rio Grande do Sul. In addition, we also included one *R*. *sanguineus* group population from Uruguay and one from Argentina. These two populations were previously assigned to the ‘temperate species’ [6], and are also from areas where canine infection by *E*. *canis* has never been properly documented [35,36,37].

## Materials and Methods

### Ethics statement

This study has been approved by the Institutional Animal Care and Use Committee (IACUC) of the Faculty of Veterinary Medicine of the University of São Paulo (protocol 2100/2010). Rabbits were purchased from a commercial breeder (Criex, Mogi das Cruzes, São Paulo, Brazil) that produce these animals for research use only. Domestic dogs were obtained from the Department of Preventive Veterinary Medicine and Animal Health of the University of São Paulo under the coordination of one of the co-authors (MBL) of the present study. These dogs were bred for research use only.

Engorged females of the *R*. *sanguineus* group were collected from naturally infested dogs under owners’ consent in the following 4 geographical sites: São Paulo city (23°32’S, 46°38’W), state of São Paulo, southeastern Brazil (designated as BSP ticks); Cachoeira do Sul (30°02’S, 52°53’W), state of Rio Grande do Sul, southern Brazil (designated as BRS ticks); Rafaela (31°15’S, 61°29’W), Santa Fé Province, Argentina, designated as ARG ticks; and Montevideo (34°51’S, 56°10’W), Uruguay (designated as URU ticks). No specific permissions were required at the tick collection locations because *R*. *sanguineus* group ticks are unpleasant pests affecting domestic dogs in South America. These tick collections did not involve endangered or protected species. According to genetic data previously reported [6], BSP ticks correspond to the ‘tropical species’, whereas BRS, ARG, and URU ticks correspond to the ‘temperate species’. All engorged females were brought to the laboratory and allowed to lay eggs in an incubator set at 27°C, 85% RH and scotophase. Part of the hatched larvae were used for the experimental infestations on dogs described below, whereas another part of the larvae were fed on tick-naïve rabbits, as previously described [38], with the purpose of obtaining unfed nymphs to be used in the experimental infestations on dogs described below. During the study, larval, nymphal, or adult infestations consisted of ≈1,000, 300–500, or 100 ticks per host, respectively, while off-host developmental stages were held under the same conditions stated previously.

### Dogs

For the present study, a total of 21 tick-naïve Beagle dogs were used. All dogs were provided by the animal facility of the Department of Preventive Veterinary Medicine and Animal Health of the University of São Paulo, where they were reared with no contact with ticks and under strict sanitary control. During the experiment, each dog was held individually within an enclosure (3.5 m x 2.5m). Two thirds of the enclosure had an open top in order to provide direct sunlight. Dogs were provided with water and a standard dry food diet (Pedigree Vital- Pro, Mars Brasil, Guararema, SP, Brazil) ad libitum, and environmental enrichment with dog toys. One week before experimental infestations, all dogs were shown to be negative for *E*. *canis* infection by both direct (*E*. *canis*-specific real-time PCR on blood sample) and indirect (serologic testing against *E*. *canis* antigens) diagnostic methods, using the protocols described below. Tick infestations on dogs were always performed inside cotton sleeves (10 to15 cm diameter) glued to the shaved back, as previously described [39]. During tick infestations, each dog received an Elizabethan collar in order to avoid removal of the cotton sleeve. Each infested dog had its cotton sleeve(s) opened daily for collection of ticks that had completed engorgement and had naturally detached.

During the experiment, medical routine checks were undertaken daily to monitor the overall health of the animals. No significant abnormality other than fever was observed. All *E*. *canis*-infected dogs recovered without clinical complications after doxycycline therapy (10 mg/Kg, 12/12 h P.O., for 28 days). Several weeks later, these animals were certified to be free of *E*. *canis* infection (tested by PCR and serology), and were sent for donation to accredited owners, together with the remaining dogs used in the present study.

### Ehrlichia canis inoculum

For experimental infection of dogs with *E*. *canis*, we used frozen infected blood derived from a dog previously inoculated with the Jaboticabal strain of *E*. *canis*, as previously described [40]. Each canine inoculation consisted of defrosting 5 mL of infected blood in a water bath at 37°C, and subsequent intravenous inoculation of the dog. For the present study, we used a stock of *E*. *canis* (Jaboticabal strain) that has been maintained in the laboratory exclusively through canine passages, with no in vitro culture passage. This *E*. *canis* strain has been shown to be highly pathogenic for dogs that were experimentally infected via infected blood [40,41].

### Molecular analyses

Ticks (unfed nymphs and adults) were processed individually for PCR as described previously using the guanidine isothiocynanate-phenol technique, as previously described [42], while canine blood samples (200 μL) were submitted to DNA extraction using the DNeasy Tissue Kit (QIAGEN, Chatsworth, CA, USA). Extracted DNA samples were tested for the presence of *E*. *canis* DNA by a real-time PCR assay using primers forward (5’-TTG CAA AAT GAT GTC TGA AGA TAT GAA ACA-3’) and reverse (5’-GCT GCT CCA CCA ATA AAT GTA TCY CCT A-3’), and the *E*. *canis*-specific taqman probe FAM-5’-AGC TAG TGC TGC TTG GGC AAC TTT GAG TGA A-3’BHQ-1, as previously described [43]. Primers and probe sequences correspond to portions of the *dsb* gene of *E*. *canis*. Because the *dsb* gene was shown to be highly polymorphic between *Ehrlichia* species, the above taqman PCR protocol was previously validated to be 100% specific for *E*. *canis* [43].

### Serological testing

Individual canine serum samples were tested by the indirect immunofluorescence assay (IFA) using *E*. *canis*-infected DH82 cells as antigen, performed with the São Paulo strain of *E*. *canis* from Brazil [44]. Reactions were performed with fluorescein-conjugated anti-dog IgG (Sigma-Aldrich, St. Louis, MO). Serum was considered to contain antibodies reactive to *E*. *canis* if it displayed a reaction at the 1:80 dilution [45]. In each slide, a serum previously shown to be nonreactive (negative control) and a known reactive serum (positive control) were tested at the 1:80 dilution. Samples that reacted at the screening dilution (1:80) were then titrated using serial two-fold dilutions to determine endpoint titers.

### Tick transmission trial I: larval acquisition of *E*. *canis* followed by vector competence of nymphs

One dog (Dog 1), intravenously inoculated with *E*. *canis* at day 0, had its rectal temperature measured daily for 30 days. Fever (rectal temperature >39.5°) occurred from 14 to 22 days after inoculation (DAI). A blood sample collected at fever onset (14 DAI) revealed *E*. *canis* DNA by real-time PCR. On the same day, four independent cotton sleeves were glued to the shaved back of the dog. On the following day (15 DAI), each of the four cotton sleeves received unfed larvae of a tick population (BSP, BRS, ARG, or URU). Engorged larvae recovered from the four cotton sleeves were held in the incubator for molting to nymphs. All procedures described for this *E*. *canis*-inoculated dog were repeated in parallel with a non-inoculated dog (Dog 2), as an experimental control. Both dogs were tested by IFA at days 0, 14 and 28.

From each of the four tick populations (BSP, BRS, ARG, or URU), a sample of 100 unfed nymphs at ≈30 days after molting were tested by real-time PCR for *E*. *canis* DNA. Samples of unfed nymphs that molted from the engorged larvae that had fed on the *E*. *canis*-infected dog were used to infest tick-naïve dogs. In this case, four dogs were infested, each with nymphs from a tick population, i.e., Dog 3 with BSP nymphs, Dog 4 with BRS nymphs, Dog 5 with ARG nymphs, Dog 6 with URU nymphs. Another dog (Dog 7) was infested with nymphs that molted from the engorged larvae that had fed on the uninfected control dog (Dog 2). In this case, nymphs from each tick population fed inside one of four cotton sleeves glued to the shaved dorsum of this fifth dog. The number of engorged ticks recovered from each cotton sleeve were recovered and counted.

**Fig 1:**
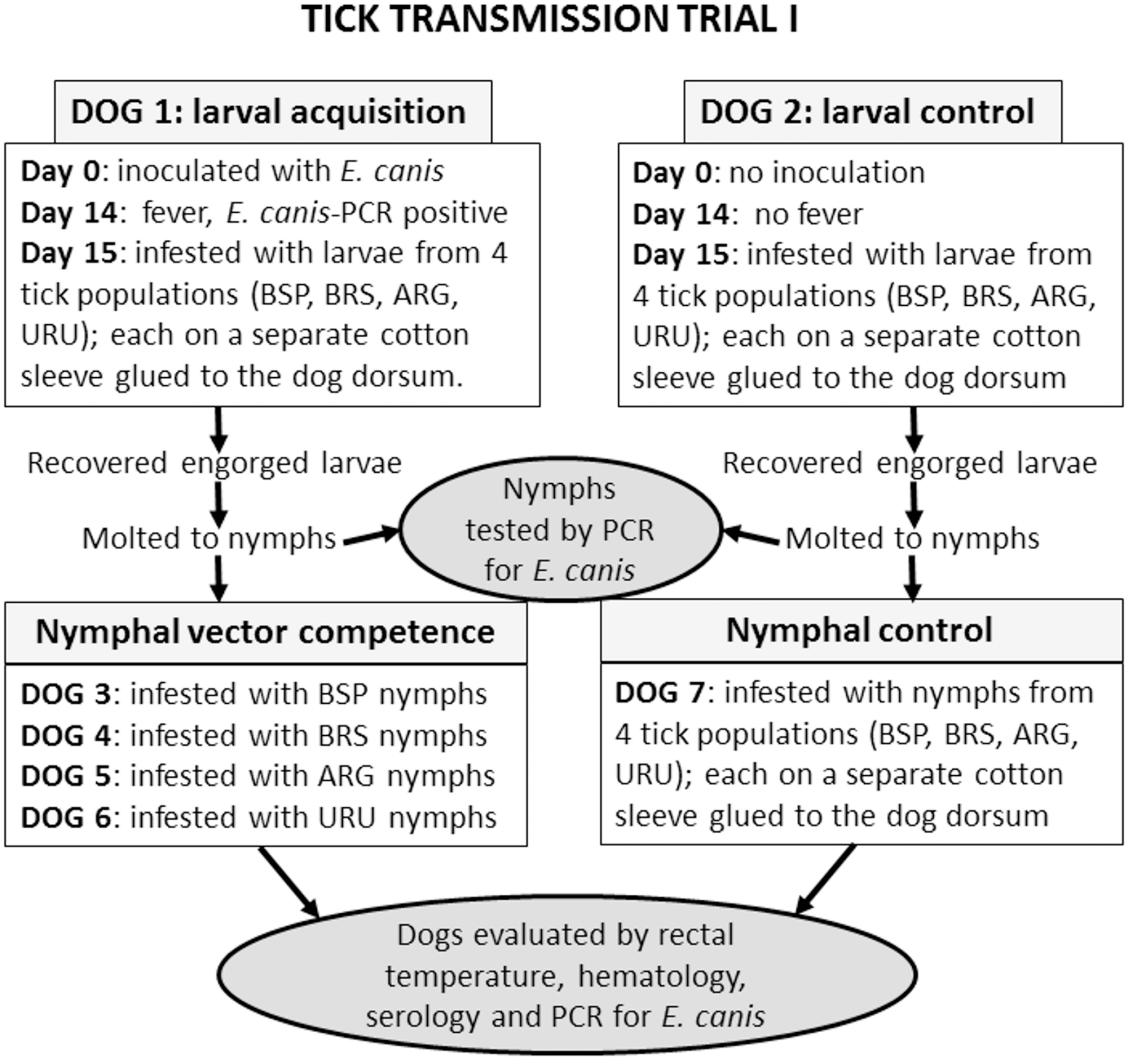
Diagram illustrating experimental procedures of tick transmission trial I in the present study.

All dogs were monitored by daily rectal temperature for 63 consecutive days post infestation, and by real-time PCR, IFA and hematology using blood samples collected at 7-day intervals. For hematology, blood cells were counted in an automatic counter (Horiba ABX Brasil, São Paulo, Brazil) to determine total number of red blood cells, white blood cells and platelets, hemoglobin level, and globular volume. Procedures described for this trial are summarized in Fig 1.

### Tick transmission trial II: nymphal acquisition of *E*. *canis* followed by vector competence of adults

One dog (Dog 8), intravenously inoculated with *E*. *canis* at day 0, had its rectal temperature measured daily for 30 days. Fever (rectal temperature >39.5^°^) occurred from 14 to 24 DAI. A blood sample collected at fever onset (14 DAI) revealed *E*. *canis* DNA by real-time PCR. On the same day, four independent cotton sleeves were glued to the shaved back of the dog. On the following day (15 DAI), each of the four cotton sleeves received uninfected unfed nymphs of a tick population (BSP, BRS, ARG, or URU). Engorged nymphs recovered from the four cotton sleeves were held in the incubator for molting to adults. All procedures described for this *E*. *canis*-inoculated dog were repeated in parallel with a non-inoculated dog (Dog 9), as an experimental control. Both dogs were tested by IFA at days 0, 14 and 28.

From each of the four tick populations (BSP, BRS, ARG, or URU), a sample of 100 unfed adults at ≈30 days after molting were tested by real-time PCR for *E*. *canis* DNA. Samples of unfed adults that molted from the engorged nymphs that had fed on the *E*. *canis*-infected dog were used to infest tick-naïve dogs. In this case, four dogs were infested, each with adults from a tick population, i.e., Dog 10 with BSP adults, Dog 11 with BRS adults, Dog 12 with ARG adults, Dog 13 with URU adults. Another dog (Dog 14) was infested with adults that molted from the engorged nymphs that had fed on the uninfected control dog (Dog 9). In this case, adult ticks from each tick population fed inside one of four cotton sleeves glued to this fifth dog. The number of engorged ticks recovered from each cotton sleeve were recovered and counted.

**Fig 2:**
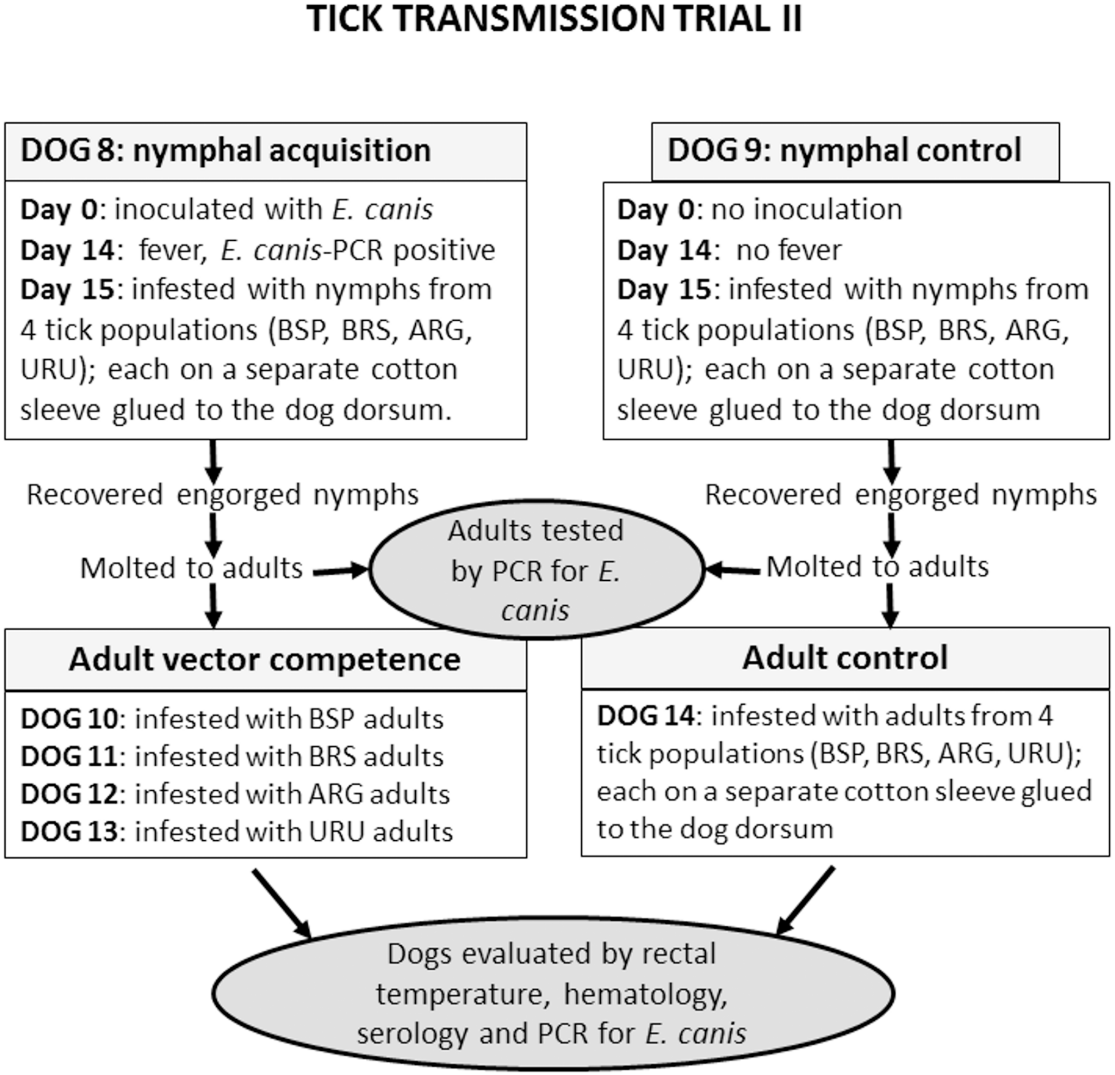
Diagram illustrating experimental procedures of tick transmission trial II in the present study.

Every dog infested with adult ticks was monitored during 63 consecutive days for rectal temperature, real-time PCR, IFA, and hematology, as described in tick transmission trial I. Procedures described for trial II are summarized in Fig 2.

### Tick transmission trial III: nymphal acquisition of *E*. *canis* followed by vector competence of adults

**Fig 3:**
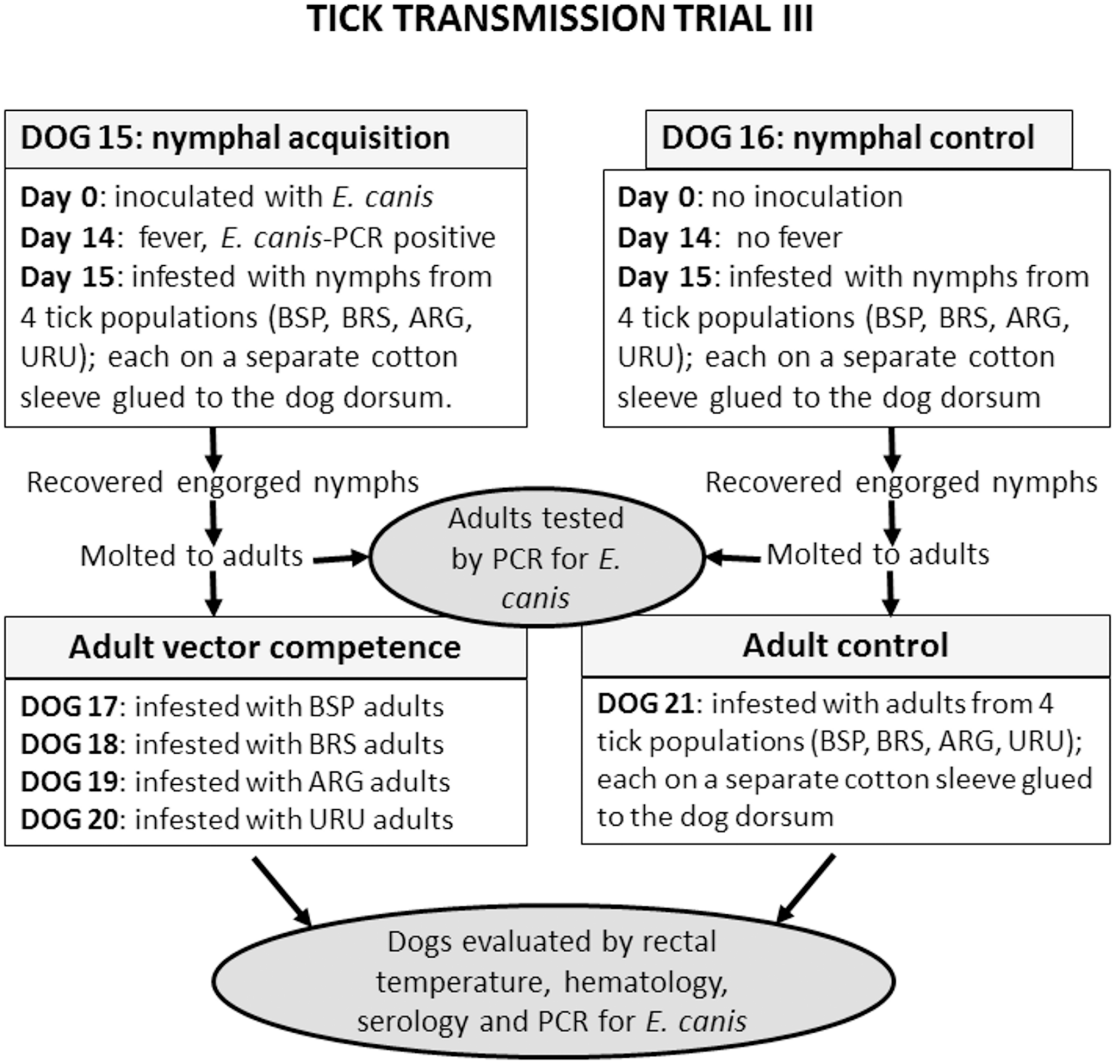
Diagram illustrating experimental procedures of tick transmission trial III in the present study.

This trial was a replication of trial II. Therefore, all procedures performed in trial II were repeated in trial III, by using other tick-naïve dogs (Dogs 15 to 21) (Fig 3). The only difference was that unfed adults, derived from engorged nymphs of the four tick populations that had fed on the *E*. *canis*-infected blood (Dog 15), were tested by real-time PCR for *E*. *canis* DNA at 7 days post molting. In addition, another group of adults of BSP and BRS ticks were tested at 30 days post molting, and another group of BRS adults were tested at 90 days post molting.

## Results

### *Ehrlichia canis* acquisition by dogs and ticks

All animals (Dogs 1, 8, 15) that were intravenously inoculated with *E*. *canis*-infected blood developed signs of CME, which started at the 14^th^ DAI and lasted until at least the 22th DAI, when doxycycline therapy (10 mg/Kg, 12/12 h P.O., for 28 days) was initiated in order to prevent clinical complications. These dogs were infested by uninfected larvae (Dog 1) or nymphs (Dogs 8 and 15) at the 15 DAI. Because larval and nymphal feeding lasted from 3 to 6 days, all engorged ticks fed during the febrile period, before the beginning of doxycycline therapy. Blood samples from the three dogs were collected and tested positively for *E*. *canis* by real-time PCR at 14 and 21 DAI, which corresponded to the day before tick infestation and the day of detachment of the last engorged tick, respectively. Through IFA, the three dogs were serologically non-reactive (titer <80) to *E*. *canis* at DAI 0. Thereafter, their IFA titers were 640 or 1,280 at DAI 14, and 10,240 or 20,480 at DAI 28. In parallel, the control dogs of the three tick transmission trials (Dogs 2, 9, 16) were always afebrile, and negative by real-time PCR and IFA (titer <80) throughout the experimental period.

In trial I, a total of 650 to 890 engorged larvae of each of the four tick populations were recovered from Dog 1 (*E*. *canis*-infected) or Dog 2 (uninfected control dog). At least 80% of these larvae successfully molted to viable nymphs. Real-time PCR results were negative for *E*. *canis* in all tiks, except for 1% of nymphs tested from BSP that had fed as larvae on Dog 1 (Table 1).

**Table 1:** Real-time PCR results of unfed ticks (nymphs or adults) after molting from ticks (engorged larvae or nymphs, respectively) that had fed on *Ehrlichia canis*-infected dogs (Dogs 1, 8 or 15) or on uninfected control dogs (Dogs 2, 9 or 16).

Tick transmission trial	Tick acquisition feeding	Tick population	Real-time PCR results for *E*. *canis* on molted ticks*		
			Tick stage	Days post molting	No. positive (%)
I	Fed as larvae on the *E*. *canis*-infected dog 1	BSP	Nymph	30	1 (1.0)
		BRS		30	0 (0)
		ARG		30	0 (0)
		URU		30	0 (0)
I	Fed as larvae on the uninfected dog 2	BSP	Nymph	30	0 (0)
		BRS		30	0 (0)
		ARG		30	0 (0)
		URU		30	0 (0)
II	Fed as nymphs on the *E*. *canis*-infected dog 8	BSP	Adult	30	7 (7)
		BRS		30	0 (0)
		ARG		30	0 (0)
		URU		30	0 (0)
II	Fed as nymphs on the uninfected dog 9	BSP	Adult	30	0 (0)
		BRS		30	0 (0)
		ARG		30	0 (0)
		URU		30	0 (0)
III	Fed as nymphs on the *E*. *canis*-infected dog 15	BSP	Adult	7	46 (46.0)
		BRS		7	16 (16.0)
		ARG		7	0 (0)
		URU		7	0 (0)
		BSP		30	27 (31.8)
		BRS		30	0 (0)
		BRS		90	0 (0)
III	Fed as nymphs on the uninfected dog 16	BSP	Adult	7	0 (0)
		BRS		7	0 (0)
		ARG		7	0 (0)
		URU		7	0 (0)

*In all cases, 100 molted ticks were tested, except for BSP adults 30 days post molting in trial III, from which only 85 adult ticks were available for testing.

In trial II, a total of 245 to 280 engorged nymphs of each of the four tick populations were recovered from Dog 8 (*E*. *canis*-infected) or Dog 9 (uninfected control dog). At least 90% of these nymphs successfully molted to viable adults. Real-time PCR results were negative for *E*. *canis* in all ticks, except for 7% of the adults tested from BSP that had fed as nymphs on Dog 8 (Table 1).

In trial III, a total of 305 to 455 engorged nymphs of each of the four tick populations were recovered from Dog 15 (*E*. *canis*-infected) or Dog 16 (uninfected control dog). At least 90% of these nymphs successfully molted to viable adults. At 7 days post molting, real-time PCR results were negative for *E*. *canis* in all ticks, except for 46% of the adults tested from BSP and 16% of the adults tested from BRS that had fed as nymphs on Dog 15 (Table 1). At 30 days post molting, 31.8% of the BSP ticks were positive, and all BRS adults were negative. At 90 days post molting, BRS adult ticks remained negative.

### Vector competence of nymphs (trial I)

Four dogs (Dogs 3, 4, 5, and 6) were infested by nymphs that had fed as larvae on the *E*. *canis*-infected Dog 1 (Fig 1). Each dog received nymphs from a single tick population, namely BSP, BRS, ARG, or URU. The number of engorged nymphs recovered per dog varied from 185 to 269 (Table 2). All dogs were clinically evaluated for 63 days after infestation. During this period, they remained afebrile (measure daily), negative by real-time PCR for *E*. *canis* (tested weekly), seronegative to *E*. *canis* (tested weekly), and had their hematological values within the normal range of health dogs (S1 Table). Dog 7 (control dog), which was infested with nymphs of the four tick populations that had fed as larvae on the uninfected control Dog 2, also remained afebrile, PCR negative, seronegative, and with hematological values within the range of healthy dogs.

**Table 2:** Results of the vector competence experiments of four populations of *Rhipicephalus sanguineus* sensu lato (BSP, BRS, ARG, URU) for *Ehrlichia canis* transmission to susceptible dogs.

Dog No.	Tick infestation data				Canine data during 63 days after tick infestation						
	Tick population	Tick stage	No. ticks infested per tick feeding cotton sleeve	No. ticks that engorged	PCR for *E*. *canis*	Seroconvertion to *E*. *canis*	Clinical data				
							Fever	Low hemoglobin concentration	Low package cell volume	Low erythrocyte count	Low platelet counts
3	BSP	Nymph ^a^	300	269	-	-	-	-	-	-	-
4	BRS			185	-	-	-	-	-	-	-
5	ARG			219	-	-	-	-	-	-	-
6	URU			250	-	-	-	-	-	-	-
10	BSP	Adult ^b^	100	68 ^d^	+	+	-	+	+	+	+
11	BRS			58 ^d^	-	-	-	-	-	-	-
12	ARG			72 ^d^	-	-	-	-	-	-	-
13	URU			59 ^d^	-	-	-	-	-	-	-
17	BSP	Adult ^c^	100	71 ^d^	+	+	-	+	+	+	+
18	BRS			75 ^d^	-	-	-	-	-	-	-
19	ARG			61 ^d^	-	-	-	-	-	-	-
20	URU			74 ^d^	-	-	-	-	-	-	-

^*a*^ Nymphs were exposed to *E*. *canis* infection by feeding as larvae on the *E*. *canis*-infected Dog 1 (trial I).

^*b*^ Adult ticks were exposed to *E*. *canis* infection by feeding as nymphs on the *E*. *canis*-infected Dog 8 (trial II).

^*c*^ Adult ticks were exposed to *E*. *canis* infection by feeding as nymphs on the *E*. *canis*-infected Dog 15 (trial III).

^*d*^ Refer to naturally detached engorged females, plus male ticks that fed on the dogs until the natural detachment of the last engorged female.

-: negative or absent

+: positive or present

### Vector competence of adults (trial II)

Four dogs (Dogs 10, 11, 12, and 13) were infested by adults that had fed as nymphs on the *E*. *canis*-infected Dog 8 (Fig 2). Each dog received adults from a single tick population, namely BSP, BRS, ARG, or URU. The number of engorged females recovered per dog varied from 21 to 34. When the last female completed engorgement (between 8 to 9 days after infestation), all male ticks were manually removed from the dogs. The number of male ticks recovered per dog varied from 31 to 48. All dogs were clinically evaluated for 63 days after infestation. During this period, all dogs remained afebrile, and all but one dog remained negative by real-time PCR for *E*. *canis*, seronegative to *E*. *canis*, and had their hematological values within the normal range of health dogs. The only exception was Dog 10 (infested with BSP adults), which became PCR-positive at 14 days after tick infestation, and remained PCR-positive at weekly intervals until 63 days after infestation, when trial II was ended (Table 2). During this period, Dog 10 showed marked alterations in its hematological values, namely hemoglobin concentration, package cell volume, erythrocyte and platelet counts below the reference range for healthy dogs (S1 Table). Dog 10 seroconverted to *E*. *canis*, as its IFA endpoint titers to *E*. *canis* were <80 at day 0, 160 at day 14, 2,560 at day 21, and 20,480 to 40,960 through days 28 to 63 after infestation. During this 63-day period, Dog 10 presented no fever, since its rectal temperature was always below 39.5°C.

Dog 14 (control dog), which was infested with adults of the four tick populations that had fed as nymphs on the uninfected control Dog 9, remained afebrile, PCR negative, seronegative, and with hematological values within the range of health dogs.

### Vector competence of adults (trial III)

Four dogs (Dogs 17, 18, 19, and 20) were infested by adults that had fed as nymphs on the *E*. *canis*-infected Dog 15 (Fig 2). Each dog received adults from a single tick population, namely BSP, BRS, ARG, or URU. The number of engorged females recovered per dog varied from 31 to 40. When the last female completed engorgement (between 8 to 9 days after infestation), all male ticks were manually removed from the dogs. The number of male ticks recovered per dog varied from 31 to 35. All dogs were clinically evaluated for 63 days after infestation. During this period, all dogs remained afebrile, and all but one dog remained negative by real-time PCR for *E*. *canis*, seronegative to *E*. *canis*, and had their hematological values within the normal range of health dogs. The only exception was Dog 17 (infested with BSP adults), which became PCR-positive at 14 days after tick infestation, and remained PCR-positive at weekly intervals until 63 days after infestation, when trial III was ended (Table 2). During this period, Dog 17 showed marked alterations in its hematological values, namely hemoglobin concentration, package cell volume, erythrocyte and platelet counts below the reference range for healthy dogs (S1 Table). Dog 17 seroconverted to *E*. *canis*, as its IFA endpoint titers to *E*. *canis* were <80 at day 0, 320 at day 14, 10,240 at days 21 and 28, and 20,480 to 5,120 through days 28 to 63 after infestation. During this 63-day period, Dog 17 presented no fever.

Dog 21 (control dog), which was infested with adults of the four tick populations that had fed as nymphs on the uninfected control Dog 16, remained afebrile, PCR negative, seronegative, and with hematological values within the range of health dogs.

## Discussion

We evaluated the vector competence of four geographic distinct populations of *R*. *sanguineus* group for the canine pathogen *E*. *canis*. In each of the three trials, acquisition feeding of ticks from the four populations were performed simultaneously on the same *E*. *canis*-infected dog. Therefore, we annulled the interference of host factors on this acquisition feeding process. The fact that we used an *E*. *canis* strain that has never been subjected to in vitro culture is also noteworthy, since it was previously reported that passages of *E*. *canis* in cell culture adversely affected its transmissibility by *R*. *sanguineus* group ticks [16].

Of the four groups of *R*. *sanguineus* tested for vector competence of *E*. *canis* (BSP, BRS, ARG and URU), only the BSP population was shown to be competent vector of *E*. *canis*, i.e., only the dogs infested with BSP adult ticks developed clinical illness, seroconverted to *E*. *canis*, and yielded *E*. *canis* DNA by PCR. This result, demonstrated by two independent replications (trials II and III), is congruent with epidemiological data, since BSP ticks were derived from the state of São Paulo, southeastern Brazil, where CME is highly endemic [25,46,47]. On the other hand, BRS ticks were derived from Rio Grande do Sul, the southernmost state of Brazil, where CME has never been properly documented. In the state of Rio Grande do Sul, canine serosurveys using *E*. *canis* antigens have reported 0 to 4.8% seropositive dogs [24,30,31,32]. When these reactive sera were titrated by IFA, they were generally characterized by low endpoint titers to *E*. *canis* [31,32]. A recent study in Rio Grande do Sul performed molecular analysis on 199 dogs for *E*. *canis* and *Anaplasma platys*. Whereas all dogs were negative for *E*. *canis* DNA, 14.07% contained *A*. *platys* DNA [24]. Because there is variable serologic cross-reactivity among *E*. *canis* and *A*. *platys* [48], it is possible that the low number of reactive dogs to *E*. *canis* antigens, previously reported in Rio Grande do Sul, could be a result of cross-reactivity to *A*. *platys*. The low *E*. *canis*-endpoint titers of these dogs [31,32] support this assumption.

In Uruguay, molecular detection of *E*. *canis* has never been reported. In one study, authors failed to detect ehrlichial DNA on a sample of 199 *R*. *sanguineus* group ticks collected from dogs [35]. In Argentina, two studies from different areas evaluated by molecular analyses 52 and 56 canine blood samples for *Anaplasmataceae* agents. Whereas all samples were negative for *Ehrlichia* agents, 13.5% and 37.5% of the dogs, respectively, were positive for *A*. *platys* [36,37]. Conversely, a recent study reported for the first time the molecular detection of *E*. *canis* DNA in the blood of 6 dogs from Argentina [49]. However, this report relied solely on 16S rRNA short sequences (318 nucleotides) and did not match 100% to any *E*. *canis* sequence available in GenBank. Because the 16S rDNA gene is highly conserved among *Anaplasmataceae* agents [50], this report requires confirmation.

In the trial II of present study, real-time PCR detected *E*. *canis* DNA in 7% of the unfed BSP adults 30 days post-molting, whereas all BRS, ARG, and URU ticks were negative by PCR. This result was corroborated by the competence vector evaluation, since only the dog infested by BSP adult ticks became infected by *E*. *canis*. In the trial III, similar results were obtained, since real-time PCR detected *E*. *canis* DNA in 31.8% of the unfed BSP adults and in none of the BRS, ARG, and URU adult ticks at 30 days post-molting. Again, only the dog infested by BSP adult ticks became infected by *E*. *canis*. On the other hand, when adult ticks were tested by real-time PCR at 7 days post-molting, 46.0 and 16.0% of BSP and BRS ticks, respectively, contained *E*. *canis* DNA. Since BRS ticks were shown to contain no *E*. *canis* DNA at 30 and 90 days post-molting, and because the dog infested with BRS ticks during trial III did not become infected by *E*. *canis*, we speculate that the 16% *E*. *canis*-positive ticks at 7 days post-molting was a result of residual *E*. *canis* DNA from the nymphal feeding in the tick body, which was not sufficient to cause an active infection in order to make the tick a competent vector of *E*. *canis*. This assumption is supported by a recent study that have successfully detected host DNA traces in at least 50% of post-molting ticks collected from the vegetation [51], which confirms that ticks are able to retain viable DNA for limited amount of time, acquired during blood feeding from a previous developmental stage. Finally, these results reinforce that molecular analyses alone does confirm vector competence of ticks for any investigated pathogen, i.e., experimental infestations on susceptible hosts must be done to confirm vector competence.

In trial I, where we tested the vector competence of *R*. *sanguineus* group nymphs for *E*. *canis*, none of the dogs became infected by *E*. *canis*, besides at least 1% of the BSP nymphs contained *E*. *canis* DNA at 30 days post-molting. While previous studies demonstrated that *R*. *sanguineus* group nymphs were able to transmit *E*. *canis* after a larval acquisition feeding, successful transmission was generally higher for adults than for nymphs [14,16]. The reasons for this variable vector competence of nymphs are unknown. However, it could be simply related to lower *E*. *canis*-infection rates among unfed nymphs, when compared to unfed adult ticks, as observed in the BSP ticks of the present study (Table 1), i.e., the lower the *E*. *canis*-infection rate, the lower the chance of an infected tick to complete engorgement on an infested dog. Finally, the explanation for lower ehrlichial infection rates in nymphs could be the much lower amount of infected blood that is ingested during larval acquisition feeding, in contrast to nymphal acquisition feedings.

**Fig 4:**
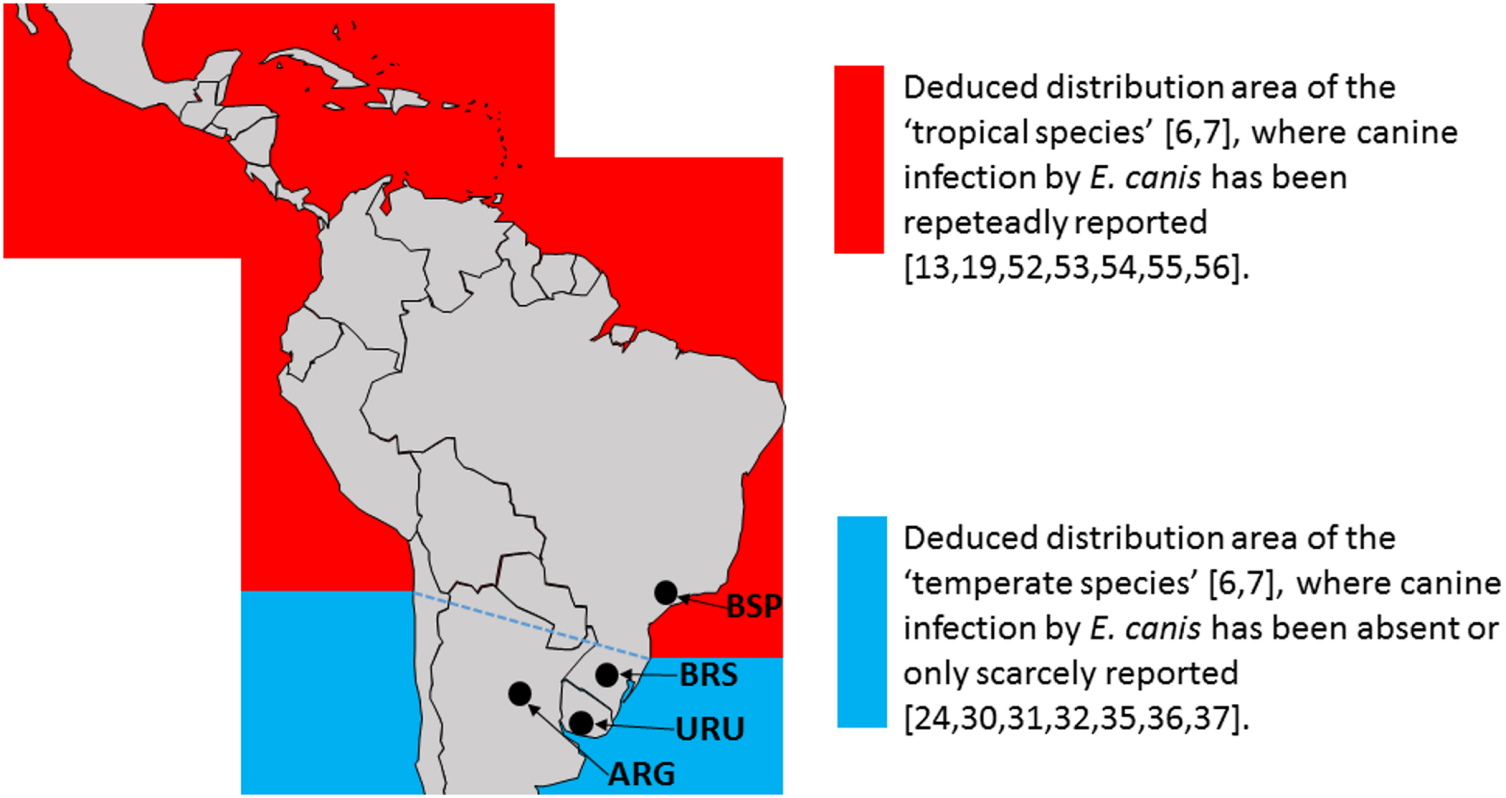
Deduced distribution area of the ‘tropical species’ and the ‘temperate species’ of *Rhipicephalus sanguineus* group in Latin America. Ticks used in the present study were BSP (from São Paulo state, Brazil), BRS (from Rio Grande do Sul, Brazil), ARG (from Argentina), and URU (from Uruguay). The map is reprinted from http://www.usgs.gov/, and edited with Microsoft PowerPoint and Paint.

The results of the present study demonstrates that, whereas BSP ticks were competent vectors of *E*. *canis*, three other populations (BRS, ARG, URU) were not. According to a recent molecular study on *R*. *sanguineus* group in Latin America [6], BSP ticks correspond to the ‘tropical species’, while BRS, ARG, and URU ticks correspond to the ‘temperate species’ (Fig 4). The tropical species was reported to occur from Mexico to Brazil (except for the southernmost part of Brazil, which includes the state of Rio Grande do Sul), where canine infection by *E*. *canis* has been widely confirmed by molecular methods [13,19,52,53,54,55,56]. On the other hand, the ‘temperate species’ was reported in the Brazilian state of Rio Grande do Sul, Argentina, Uruguay, and Chile, from areas where *E*. *canis* has never been properly reported. Based on the results of the present study, we conclude that the absence or scarcity of cases of CME due to *E*. *canis* in the southern cone of South America is a result of vector incompetence of the *R*. *sanguineus* group ticks (‘temperate species’) that prevail on dogs in this part of South America. As stated by Nava et al. [1], further studies are required to elucidate the taxonomic position of the *R*. *sanguineus* group species complex in the world, which includes at least two species in South America, herein designated as ‘tropical species’ and ‘temperate species’.

## Supporting Information

S1 Table Hematological data of the dogs that were infested with ticks during the vector competence infestations. (XLSX)
